# Transcatheter aortic valve implantation in an orthotopic heart transplant recipient with bicuspid aortic valve

**DOI:** 10.1002/ccr3.1845

**Published:** 2018-10-17

**Authors:** Subhi I. Akleh, Alykhan Bandali, Richard Edwards

**Affiliations:** ^1^ The Freeman Hospital Newcastle upon Tyne UK

**Keywords:** aortic stenosis, bicuspid aortic valve, heart transplant, TAVI

## Abstract

Increasing longevity of heart transplantation recipients and aging donor population accompanied by the older age at transplantation led to an increase in the prevalence of degenerative valvular disease in particular aortic stenosis. TAVI is considered a safe and feasible alternative compared to conventional SAVR in this high‐risk population.

## 1 INTRODUCTION

Aortic stenosis is the commonest degenerative valve disorder in the west. Transcatheter interventions have introduced a new option of valve replacement in high‐risk patients. An outstanding question is the management of degenerative valve disease in the heart transplant (HTx) population given their unique combination of comorbidity, lack of representation in studies to derive cardiothoracic surgical risk scoring and technical difficulties of redo surgery. Here, we present a 77‐year‐old man undergoing transcatheter aortic valve implantation (TAVI) for degenerative bicuspid aortic (BAV) valve stenosis 23 years after HTx.

Aortic valve stenosis (AS) is by far the commonest valvular disease in the aging population. In addition, BAV is the most prevalent congenital valvular abnormality. It is considered a significant risk factor for premature aortic valve disease, most commonly AS.^1^ BAV disease was an exclusion criterion in fundamental clinical trials to test the safety and efficacy of TAVI, largely because of the unique morphological features and associated aortic pathology of this condition.^2^, ^3^ Limited data exist for outcomes from TAVI in BAV and, despite promising single‐center series,^4^, ^5^ a recently published multi‐center series raised concerns about the excess of paravalvular regurgitation.^6^

There is no doubt that the increasing longevity of HTx recipients accompanied by the older age at transplantation and aging donor population led to an increase in the prevalence of degenerative valvular disease. This increase, in this unique population, rendered optimal treatment strategy unknown in the context of their specific comorbidity.

We present our experience of a 77‐year‐old man presenting with severe allograft bicuspid aortic stenosis 23 years after HTx.

## 2 CASE PRESENTATION

A 77‐year‐old man attended our services with exertional dyspnoea secondary to aortic valve stenosis. He received an orthotopic heart transplantation (HTx) in 1994 for idiopathic dilated cardiomyopathy (DCM). Unfortunately, we have no records of the patient's transplant operative data given the fact that his procedure was done 23 years ago. He remained asymptomatic during follow‐up except for paroxysmal atrial flutter for which he received a single chamber pacemaker in 2008 and later, atrial flutter ablation in 2010. Patient was adherent to his medication regimen and did not show any signs of transplant rejection on several cardiac biopsies. His post‐transplant cardiovascular risk factors included systemic hypertension, dyslipidaemia, and stable stage 4 chronic renal dysfunction (eGFR 23 mL/min/1.73 m^2^). Serial transthoracic echocardiography (TTE) performed in our institution showed progressive degenerative aortic valve disease.

At presentation, his TTE showed degenerative bicuspid aortic valve with fusion of the right and left coronary cusps by an incomplete raphe. The appearance of the valve was consistent with severe aortic stenosis which was confirmed by hemodynamic Doppler assessment that revealed a peak gradient of 65 mm Hg, aortic valve area of 0.9 cm^2^ derived from the continuity equation and a dimensionless velocity index (DVI) of 0.24. Left ventricular function was normal with an ejection fraction (EF) of 59% by Simpson's method. Further evaluation of the aortic valve and aorto‐iliac anatomy was pursued by a Multi‐detector computed tomography (MDCT). It confirmed the morphology of a heavily calcific BAV, the absence of associated aortopathy, and suitability for transfemoral approach. The maximal aortic annulus dimension was measured as 25 mm with an aortic root diameter of 32 mm at the level of the sinuses of Valsalva. Coronary angiography was performed to screen for cardiac allograft vasculopathy (CAV) which did not show any evidence of obstructive coronary disease.

In addition, he was noted on admission to be bradycardic with episodes of second‐degree mobitz type 2 atrio‐ventricular (AV) heart block. Electrophysiology service was consulted and decided the need to upgrade his pacemaker to a dual‐chamber system following the TAVI procedure.

His case was discussed at the Heart Valve Team meeting with a consensus that TAVI would be the optimal intervention strategy being a high‐risk surgical candidate with a Society of Thoracic Surgery (STS) predicted risk of 30 days mortality of 7.035%.

The TAVI procedure was performed according to the standard local TAVI protocol. Vascular access was obtained with ultrasound guidance under local anesthesia and conscious sedation. Heparin (6000 units) was given intraoperatively to achieve an activated clotting time (ACT) greater than 250 seconds. A balloon expandable 29 mm Edwards Sapien 3 transcatheter heart valve (Edwards Lifesciences, Irvine, CA, USA) was advanced via the right femoral artery through the calcified, transplanted native aortic valve without prior balloon aortic valvuloplasty. Final positioning was confirmed by fluoroscopic guidance. Under rapid ventricular pacing, by temporary pacing wire via the left femoral vein, expansion of the prosthesis over the stenotic valve was accomplished with excellent results and no immediate complications. The total amount of contrast used was 60 mL and subsequent renal function tests were stable. His pacemaker was electively upgraded to a dual‐chamber system the following day as planned earlier due to pre‐existing high degree heart block. Pre‐discharge TTE revealed a well‐positioned aortic valve prosthesis with a peak and mean trans‐prosthesis gradients of 14 mm Hg and 12 mm Hg respectively. There was no evidence of valvular or paravalvular regurgitation on color flow Doppler and the LV systolic function remained normal.

Patient showed immediate symptomatic and hemodynamic improvement and was discharged from hospital 48 hours post index procedure. He was maintained on his regular medication including the immunosuppressive therapy. At the routine 1‐month clinic follow‐up the patient was doing well and did not report any symptoms with no limitation of his physical activity (NYHA 1).

## 3 DISCUSSION

The first‐in‐man TAVI was performed in 2002 by Alain Cribier in France.^7^ Ever since excellent achievements have been made in the field of TAVI saving lots of lives. The PARTNER trials comparing TAVI with SAVR in high‐risk severe aortic stenosis patients were the cornerstone for TAVI, which showed similar survival rates at 1 year,^8^ improved valve hemodynamic at 2 years, reduction in symptoms, death rates, and hospitalizations.^7^, ^9^ TAVI, however, showed more frequent paravalvular regurgitation and increased late mortality.^7^, ^9^ Although TAVI has been limited to high‐risk and inoperable patients, carefully selected younger patients may benefit from this approach if advantages of the TAVI outweigh the results expected from conventional SAVR.^10^ HTx recipients with aortic valve disease may be considered eligible candidates for TAVI because of patient‐specific and procedure‐specific features.

In 2010 Seiffert et al reported the first case of TAVI in a HTx recipient. The patient was a high‐risk 81 years old who underwent a TAVI for severe AS 15 years post‐HTx due to end‐stage DCM. Pre‐operative assessment revealed a severely depressed LVEF of 30% secondary to concomitant CAV. Percutaneous coronary intervention (PCI) for an 80% stenosis of the LAD artery was carried out and TTE post intervention showed improvement of LV systolic function with an EF of 50%. The author also mentioned that the mean aortic pressure gradient was 23 mm Hg with an effective orifice area of 0.6 cm^2^ which is consistent with a paradoxical low gradient AS. The author did not clarify the underlying morphology of the AV or if further investigation was performed to confirm the presence of severe AS such as low‐dose Dobutamine stress echocardiography (DSE). Transapical approach was adopted due to extensive calcification of both ascending and descending aorta as well as kinking of the iliac arteries. A 26 mm Edward Sapien THV was implanted successfully with only trivial paravalvular regurgitation.^11^

In the same year, Bruschi et al reported a 67 years old man who had a transfemoral TAVI for severe AS that developed 9 years after his HTx. Pre‐operative TTE showed a peak transvalvular pressure gradient of 87 mm Hg, indexed AVA of 0.5 cm^2^/m^2^ and severe LV systolic dysfunction (EF 35%). A 29 mm CoreValve prosthesis (Medtronic, Minneapolis, MN) was deployed with excellent results and only mild paravalvular regurgitation which was seen on the pre‐discharge TTE.^12^

TAVI was not only utilized in high‐risk HTx recipients with severe AS but included patients with significant aortic regurgitation (AR). Zanuttini et al reported the first case of a transfemoral TAVI performed on a high‐risk 75‐year‐old HTx recipient for severe degenerative AR. The patient was transplanted 14 years earlier due to end‐stage DCM as a result of previous aortic and mitral valve replacement. The pre‐operative TTE showed mildly dilated LV, moderate LV dysfunction (EF 40%) and a thickened tri‐leaflet AV cusps. Doppler study demonstrated severe central jet of AR as a result of a deformed and retracted left coronary cusp. Similar to Bruschi et al case, a 29 mm CoreValve was used based on the decision of the heart team to proceed to TAVI for the very high‐risk profile (EuroSCORE 36%). His hospital course was complicated by a third‐degree AV block 48 hours post‐TAVI for which he received a permanent pacemaker. He also developed lower respiratory infection that was successfully treated with antibiotics. Pre‐discharge TTE showed a well functioning prosthetic valve, with peak and mean pressure gradients of 16 mm Hg and 10 mm Hg, respectively, and a mild paravalvular regurgitation.^13^

Furthermore, in 2013, Praetere et al reported a 77‐year‐old patient who received his HTx in 1993 for end‐stage ischemic cardiomyopathy. Post transplantation the patient developed severe CAV, which lead to multiple PCIs in 1996, 1997 and 2003. Despite revascularization he remained symptomatic with reduced exercise tolerance. Baseline TTE showed an AVA of 1.0 cm^2^, severely depressed LV systolic function with an EF of <25% and evidence of classical low flow, low gradient AS. Additional testing with low‐dose DSE failed to confirm the true severity of AS and a decision was to proceed with balloon aortic valvuloplasty (BAV) to evaluate its effects on symptoms and LVEF. A modest improvement in the LVEF (up to 30%) and in patients’ symptoms were noted, however, these improvements were transient and symptoms relapsed after 7 months. This transient improvement with BAV justified the decision to proceed with TAVI which was achieved using a 23 mm Edwards Sapien valve. TTE post‐TAVI demonstrated a normal functioning valve, peak and mean pressure gradients were 18 and 11 mm Hg respectively, stable LVEF of 30% and no paravalvular aortic regurgitation.^14^

Ahmad et al reported a TAVI in a 25‐year‐old female who received an urgent HTx at the age of 11 years due to severe congestive heart failure. She had corrective operations for multiple ventricular septal defects at the age of 1 and 3 years of age. Interestingly, the donor heart was from a 62‐year‐old female with pre‐existing moderate coronary artery disease and mild AS. After 14 years, she developed exercise‐limiting symptoms with a NYHA III class. TTE showed a heavily calcific tri‐leaflet AV with combination of severe AS (Peak gradient 77 mm Hg, mean gradient 44 mm Hg, AVA <1.0 cm^2^) and severe AR with a preserved LVEF. Patient underwent transfemoral TAVI with a 23 mm Edwards Sapien 3 valve with no complication. Her symptoms improved dramatically and post‐operative TTE showed normal functioning prosthesis with peak gradient of 20 mm Hg, mean gradient 11 mm Hg and no paravalvular regurgitation.^15^

In December of the same year, Herrmann et al presented a 73 years old male who had a successful transfemoral TAVI with a 26 mm Edwards Sapien 3 transcatheter heart valve 13 years post heart transplant. The pre‐operative TTE showed a severely stenosed BAV with a mean transvalvular pressure gradient of 43 mm Hg and a calculated AVA of 0.58 cm^2^. The heart valve team opted for TAVI being a high‐risk surgical candidate with a STS predicted risk of 30 days mortality of 8.024%. Post‐operative TTE revealed a peak and mean pressure gradient of 27 mm Hg and 13 mm Hg respectively with no paravalvular regurgitation.^16^

As a consequence of worldwide heart donor paucity and increased demand, marginal donors are often accepted particularly in patients with an urgent call for HTx. The marginal donor is often characterized with higher age and comorbidities such as diabetes, hypertension and even mild valvular heart disease. Survival rates after HTx have increased significantly which might increase the likelihood for development of significant age‐related valvular disease in the HTx population.

Although cardiac surgery after heart transplantation is considered a safe and effective option as stated by Goerler et al^17^ in 2010, the author also highlighted the increased risk of bleeding owing to marked adhesions after previous surgical procedures, as well as the increased risk of renal failure, infection and wound healing disorders due to long‐term immunosuppression. However, the author did not take into consideration the risks associated with general anesthesia, prolonged hospital admission, nosocomial infection and the cost‐effectiveness impact on the healthcare system which are considerably lower with TAVI.

Extensive literature search showed lack of data studying the safety and efficacy of TAVI in HTx recipients specifically in the setting of BAV disease.

## 4 CONCLUSION

TAVI has been rarely performed in HTx patients with no available data comparing TAVI versus SAVR in this isolated population. Indeed, in this case, conventional surgery would have carried a higher risk of complication of a redo surgery, profound renal failure and poor wound healing due to immunosuppressive treatment. The current paper demonstrates a successful transfemoral TAVI in a HTx recipient. To our knowledge, this is the seventh case worldwide reporting TAVI in HTx recipient, the second in a bicuspid aortic valve and the first case receiving a TAVI after 23 years post‐HTx, which is considered the longest duration reported so far. This emphasizes the need for further studies to assess safety and efficacy of TAVI in this unique population with increasing survival durations owing to the advances in medicine.

## AUTHOR CONTRIBUTION

RE: performed the TAVI procedure and supervised the project. SA: performed the post‐TAVI echocardiography and wrote the manuscript. AB: assisted in the drafting of the manuscript.
